# Xi Lei San Attenuates Dextran Sulfate Sodium-Induced Colitis in Rats and TNF-*α*-Stimulated Colitis in CACO2 Cells: Involvement of the NLRP3 Inflammasome and Autophagy

**DOI:** 10.1155/2021/1610251

**Published:** 2021-04-21

**Authors:** Zhang Tao, Xiaoqing Zhou, Yan Zhang, Wenfeng Pu, Yi Yang, Fuxia Wei, Qian Zhou, Lin Zhang, Zhonghan Du, Ji Wu

**Affiliations:** ^1^Department of Gastroenterology, Nanchong Central Hospital, The Second Clinical Medical College, North Sichuan Medical College, Nanchong, Sichuan Province, China; ^2^Department of Gastroenterology, Jing Men No. 2 People's Hospital, Jing Men City, Hubei Province, China; ^3^Department of Gastroenterology, People's Hospital of Nanjiang, Bazhong City, Sichuan Province, China; ^4^Department of Urology, Nanchong Central Hospital, The Second Clinical Medical College, North Sichuan Medical College, Nanchong, Sichuan Province, China

## Abstract

**Objective:**

Inflammatory bowel disease (IBD) is a chronic nonspecific inflammatory bowel disease with an unclear etiology. The active ingredients of traditional Chinese medicines (TCMs) exert anti-inflammatory, antitumor, and immunomodulatory effects, and their multitarget characteristics provide them with a unique advantage for treating IBD. However, the therapeutic effects and underlying mechanisms of Xi Lei San in treatment of IBD remain unknown. This study was designed to investigate whether Xi Lei San exerted an anti-inflammatory effect in IBD via a mechanism involving NLRP3 inflammasomes and autophagy.

**Methods:**

We successfully established a rat model of dextran sulfate sodium- (DSS-) induced colitis as well as a cellular model of TNF-*α*-induced colitis. Xi Lei San and indirubin were identified by HPLC analysis. Rats were treated with Xi Lei San or alum crystals, and their body weights and morphology of intestinal tissues were examined. A western blot analysis was performed to determine the expression levels of inflammasome-related proteins and autophagy-related proteins, ELISA was performed to analyze IL-1*β*, IL-18, and IL-33 concentrations, and flow cytometry was used to monitor cell apoptosis and ROS levels.

**Results:**

Xi Lei San and indirubin were identified by HPLC analysis. We found that Xi Lei San could significantly increase the weights of rats and improve the structure of the intestinal tissues in DSS-induced colitis model rats. We also found that Xi Lei San significantly inhibited NLRP3 inflammasome activity, reduced the levels of inflammatory cytokines, and suppressed autophagy in DSS-induced colitis model rats. *In vitro* experiments revealed that Xi Lei San could repress apoptosis as well as ROS and inflammatory cytokine production in TNF-*α*-induced CACO2 cells by reducing the activity of NLRP3 inflammasomes and autophagy.

**Conclusions:**

Our findings showed that Xi Lei San significantly ameliorated IBD by inhibiting NLRP3 inflammasome, autophagy, and oxidative stress.

## 1. Introduction

Inflammatory bowel disease (IBD) is a persistent, chronic, recurrent, and nonspecific inflammatory disease [1]. IBD is characterized by the development of intestinal inflammation caused by polymorphonuclear neutrophils, macrophages, lymphocytes, and mastocytes and can result in mucosal damage and ulcers [2]. The incidence of IBD has gradually increased in recent years. While the current affected population is mainly young adults, the incidence of IBD is also increasing among children [3]. IBD includes two types of intestinal idiopathic inflammatory diseases: ulcerative colitis (UC) and Crohn's disease (CD) [4]. Currently, it is generally believed that the pathogenic factors associated with IBD include intestinal mucosa damage, persistent inflammatory factors, genetic susceptibility, intestinal microecological damage, and environmental factors [5–7]. However, the etiology and specific pathogenesis of IBD remain unclear. The current treatment for IBD consists of general treatment (rest and nutrition), medications (anti-inflammatory drugs and immune regulators), and surgical treatment (total colon resection) [8, 9]. Conventional Western medicines have drawbacks that include side effects, a high financial cost, and a high rate of disease recurrence [10, 11]. Therefore, it is important to explore the pathogenic mechanism of IBD and develop new therapeutic drugs and methods for its treatment.

Numerous clinicians have achieved good results in treating diseases by using combined methods of syndrome differentiation, internal administration, external irrigation, and acupuncture [12, 13]. Previous research revealed that traditional Chinese medicines (TCMs) produce obvious effects in treating colitis and have the important advantages of low cost, fewer side effects, and ease of use for an extended time period [14, 15]. Some domestic literature has reported that Xi Lei San, a class of Chinese patent medicine, is highly effective for treating IBD and shows unique advantages in terms of symptom control and producing fewer side effects [16]. Xi Lei San consists of seven TCMs, including indigo naturalis, calculus bovis factitius, pearl, borneol, human fingernails, ivory crumbs, and uroctea, and has been shown to be effective in detoxifying saprophytes [17]. Xi Lei San was recently shown to facilitate the healing of peptic ulcers and exert broad-spectrum antibacterial, anti-inflammatory, and astringent myogenesis effects [17]. However, the efficacy of Xi Lei San and its mechanism of action in treatment of IBD are not fully understood.

The NLRP3 inflammasome is a multiprotein complex [18] that not only plays a central role in the innate immune response but also plays an important role in the pathogenesis of autoimmune diseases by regulating the differentiation and function of immune cells [19, 20]. The activation of NLRP3 inflammasomes can activate caspase-1, further induce the maturation of interleukin 1*β* (IL-1*β*), and thereby regulate an inflammatory response [21]. Some studies have shown that NLRP3 inflammasome is closely related to IBD remission [22, 23]. Additionally, autophagy has been widely recognized being participated in IBD. For example, Pott et al. suggest that autophagy in intestinal epithelial cell protects cells from chronic inflammatory injury (Pott, 2018 #52). However, the relationship between autophagy and NLRP3 inflammasomes remains uncertain whether Xi Lei San alleviates IBD by regulating NLRP3 inflammasome activity and autophagy.

In the current study, we successfully established both a DSS-induced rat model of colitis and a TNF-*α*-induced cellular model of colitis. We then explored the influence of Xi Lei San on body weight, morphological changes in intestinal tissues, NLRP3 inflammasome, autophagy, and apoptosis. Our studies revealed that Xi Lei San exerts a regulatory effect on NLRP3 inflammasomes, suggesting those inflammasomes as targets for treating IBD.

## 2. Materials and Methods

### 2.1. Animals

A total of 20 healthy SPF-grade SD female rats (180-220 g) were purchased from Sun Yat-sen University (No. 44008500019433; License no.: SYXK (Guangdong) 2016-0112) and housed at the North Sichuan Medical College. The rats were housed in a room with a 12 h light/dark cycle, a temperature of 20-24°C, and a humidity of 50-60%; food and water were available *ad libitum*. The study protocol was approved by the Ethical Review Committee of SLAS.

### 2.2. Colitis Rat Model

After 1 week of adaptive feeding, SD rats were fed 4.5% dextran sulfate sodium (DSS; MP Biomedicals) in water for 7 days along with their normal diet. In brief, the rats were randomly assigned to a control group (conventional diet), model group (regular diet followed by drinking 4.5% DSS in water for 7 days), low-dose Xi Lei San group (DSS model rats were treated with 100 mg/kg/day of Xi Lei San for 7 days), and high-dose Xi Lei San group (DSS model rats were treated with 200 mg/kg/d of Xi Lei San for 7 days). The body weights of the SD rats in each group were examined at 1, 3, 5, 7, 9, 11, and 14 days. The SD rats received a daily Disease Activity Index (DAI) score. The stool properties of the rats were observed, and any fecal occult blood was detected and recorded. After the dietary intervention, a 10 cm segment of distal colon was collected from each rat. The colon segments were quick-frozen in iquid nitrogen and subsequently stored at -80°C for use in western blot assays. A portion of each distal colon was fixed in 4% paraformaldehyde for H&E staining and immunohistochemistry analysis. Samples of blood serum were collected and stored in a -80°C refrigerator for use in ELISA.

### 2.3. Cell Culture and Treatment

Frozen CACO2 cells were warmed to 37°C and then pelleted by centrifugation (1000g for 5 min). After being resuspended, the cells were cultured in Dulbecco's modified Eagle's medium (DMEM, Invitrogen, Carlsbad, CA, USA) containing 10% fetal bovine serum (FBS; Gibco, cat. no. 10099-141) and 10 g/mL penicillin/streptomycin (Gibco, cat. no. 15140122) in a 37°C incubator containing 5% CO_2_. A cellular model of colitis was constructed by adding TNF-*α* to the CACO cells. Other groups of CACO2 were also treated with Xi Lei San and/or alum crystals in addition to TNF-*α*.

### 2.4. High-Performance Liquid Chromatography (HPLC)

As in previous studies [24, 25], the chromatograms of Xi Lei San and indirubin were confirmed by HPLC. Xi Lei San and indirubin solutions were prepared and analyzed by HPLC performed using a Hypersil NH2 column (250 mm × 4.6 mm, 5 mm). The column temperature, flow rate, and injection volume were 30°C, 1 mL/min, and 2.0 *μ*L, respectively. The detection wavelength was 289 nm. The mobile phase consisted of water (A) and acetonitrile (B), and the samples were eluted using the following gradient: 0~5.5 min, 97% (B); 5.5-6 min, 97-90% (B); 6~20 min, 90% (B); and 20~21 min, 90%~97% (B). MS/MS was conducted using ionized electrospray ionization (ESI+). The atomization temperature of the ion source was 500°C; the flow rates were 8, 6, and 14 mL/min, respectively; and the focusing voltage, suction voltage, and suction voltage of collision charge were 82 V, 10 V, and 13 V, respectively. Results were determined using a multireaction monitoring model. The Xi Lei San used for this study was provided by Zhejiang Dongtan Pharmaceutical., Ltd., Taiji Group (Batch No. 18010003).

### 2.5. Hematoxylin and Eosin Staining (H&E Staining)

Tissues were fixed by 4% paraformaldehyde and embedded by paraffin. Serial sections were obtained, and then, dehydration and dewaxing were performed. Sections were placed in distilled water for 2 min and then stained with hematoxylin for 5 min. After washing, the sections were dehydrated with 95% ethanol for 5 s and then stained with eosin for 30 s. After staining, the sections were dehydrated two times in 95% ethanol and then made transparent by two 5 min immersions in xylene. Finally, the tissue sections were sealed with neutral gum.

### 2.6. Western Blotting

The colonic tissues from each group were ground, and the CACO2 cells from each group were collected. Next, the cells were lysed in RIPA lysate buffer (Beyotime, China) containing a protease inhibitor and then centrifuged. The total protein content in each supernatant was determined using a Bradford protein assay kit (Bio-Rad, Hercules, CA, USA). A 40 *μ*g sample of total protein from each sample was separated by SDS-PAGE electrophoresis (separation gel: 80 V, spacer gel: 100 V), and the protein bands were transferred onto PVDF membranes (30 mA, 90 min). After being blocked, the membranes were incubated with primary antibodies overnight at 4°C, followed by incubation with an HRP-labeled secondary antibody for 60 min at 37°C. The immunostained protein bands were visualized using an ECL kit (Thermo Fisher Scientific, Waltham, MA, USA), and their staining intensities were determined. The primary antibodies used were obtained from Abcam (Cambridge, UK) and included antibodies against NLRP3 (1 : 1000, ab263899), IL-1*β* (1 : 1000, ab9722), caspase-1 (1 : 1000, ab62698), ASC (1 : 1000, ab175449), IL-18 (1 : 1000, ab71495), LC3B (1 : 800, Boster), p62 (1 : 1500, Boster), and GAPDH (1 : 2000, ab9485).

### 2.7. ELISA

The levels of IL-1*β*, IL-18, and IL-33 were determined by using the appropriate ELISA kits according to instructions provided by the supplier. The optical density (OD) of each well at 450 nm was determined by using an automatic enzyme marker (RT-2100C).

### 2.8. Flow Cytometry Analysis for Apoptosis

The CACO2 cells from each group were suspended in PBS and then diluted to a concentration of 1 × 10^6^ cells/mL. After the cells were washed, 5 *μ*L of PI and 5 *μ*L of Annexin V-FITC (Abnova, cat. no. KA3805) were added to each tube of cells, and the tubes were placed in the dark for 10 min. Next, 200 *μ*L of binding buffer was added to each tube, and the cells were analyzed by flow cytometry (BD Biosciences, Franklin Lakes, NJ, USA). The data were analyzed using ModFit software.

### 2.9. Immunohistochemistry

Serial sections have undergone antigen retrieval and blocking after dehydration and dewaxing. Subsequently, sections were incubated with primary antibody (caspase-3, Boster Bio, cat. no. PB9188, 1 : 100). Afterward, sections were incubated with secondary antibody (Dako, cat. no. K5007, ready-to-use) followed by color development using DAB reagent (Beyotime, cat. no. P0202) and redyed by hematoxylin. Finally, sections were sealed by neutral resins for observation under a microscope.

### 2.10. Flow Cytometry Analysis for ROS

Treated CACO2 cells were placed into the wells of 6-well plates with ~80% surface coverage. After 48 h of culture, the cells in each well were washed 3 times and then treated with 40 *μ*mol/L DCFH-DA staining solution for 30 min. After washing, the ROS levels were determined by flow cytometry (BD Biosciences) performed with an excitation wavelength of 488 nm and an emission wavelength of 525 nm.

### 2.11. Statistical Analysis

All data were analyzed using IBM SPSS Statistics for Windows, Version 20.0 software (IBM Corp., Armonk, NY, USA), and results are expressed as the mean value obtained from three replicate experiments. Differences between groups were analyzed by one-tailed ANOVA followed by Tukey's post hoc test. *P* values < 0.05 were considered to be statistically significant.

## 3. Results

### 3.1. Xi Lei San and Indirubin Were Identified by HPLC

**Figure 1:**
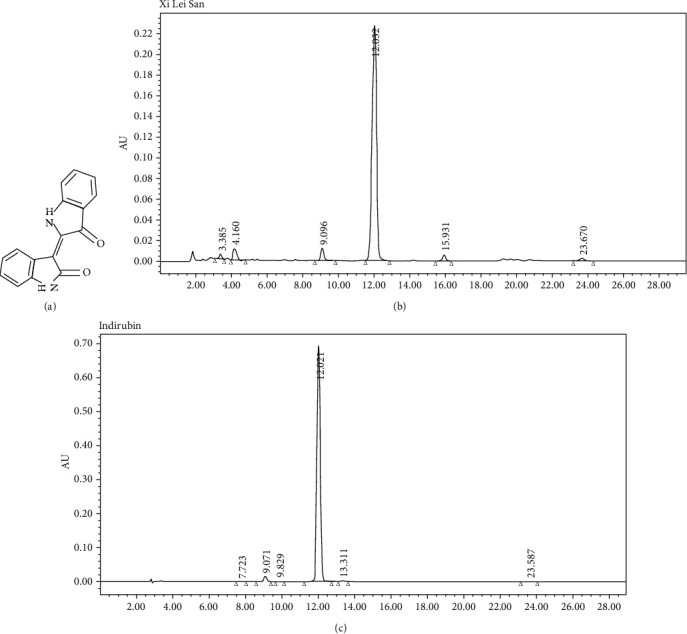
Xi Lei San and indirubin were identified by HPLC. (a) The molecular structure of indirubin. The chromatograms of (b) Xi Lei San and (c) indirubin as obtained by HPLC.

To examine the potential effects of Xi Lei San in colitis, we first analyzed the structure and molecular weight of Xi Lei San and indirubin. The molecular structure of indirubin is shown in Figure 1(a), and its molecular formula is C_16_H_10_N_2_O_2_. We used HPLC to confirm the chemical structures and relative molecular weights of Xi Lei San and indirubin used in this study. A chromatographic peak for indirubin was detected, as shown in Figure 1(b). The same method was used to detect the chromatographic peak for Xi Lei San (Figure 1(c)). These findings showed that indirubin was the main active component.

### 3.2. Xi Lei San Relieved DSS-Induced Colitis in Rats

**Figure 2:**
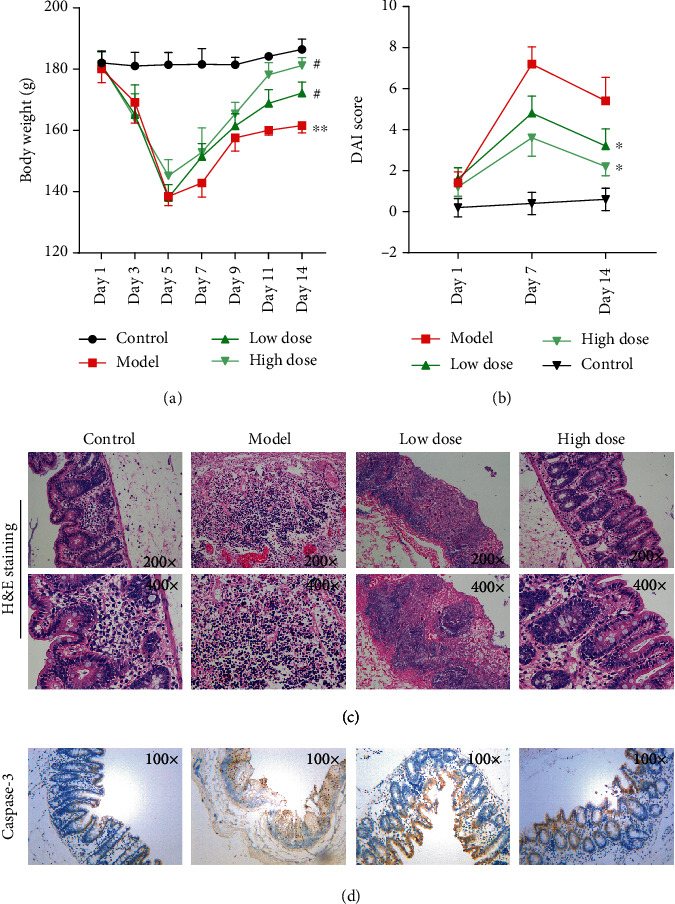
Xi Lei San relieved DSS-induced colitis in rats. (a) A DSS-induced rat model of colitis was constructed, and the model rats were treated with low or high doses of Xi Lei San. The body weights of the rats in each group were measured. (b) After treatment with Xi Lei San, a DAI score was calculated for each group of rats. (c) The pathological examination for rat intestinal tissues was observed after H&E staining. (d) Caspase-3 expression was detected by using immunohistochemistry. ^∗^*P* < 0.05 and ^∗∗^*P* < 0.01 vs. the control group; ^#^*P* < 0.05 vs. the model group.

In order to assess the therapeutic effect of Xi Lei San in colitis, we established a DSS-induced model of enteric colitis in rats. We found that administration of 4.5% DSS for 7 days resulted in a significant weight loss in rats, and the weight loss could be attenuated by the addition of Xi Lei San and especially high doses of Xi Lei San to the diet of the model rats (*P* < 0.05 and *P* < 0.01, Figure 2(a)). We also found that the DAI values for the DSS model rats were significantly reduced after treatment with Xi Lei San, due to increases in body weight reduced defecation (*P* < 0.05, Figure 2(b)). H&E staining results showed that the intestinal mucosa of rats in the normal group was intact and no obvious abnormalities were observed. In the DSS model group, most of the intestinal glands had disappeared, the structure of epithelial cells was destroyed, and the intestinal tissues showed severe inflammatory changes and evidence of neutrophil infiltration. However, Xi Lei San notably ameliorated all those changes in the DSS model group (Figure 2(c)). Besides, caspase-3 expression was higher in the DSS model group compared with that in rats in the control group, while Xi Lei San administration reversed caspase-3 expression (Figure 2(d)). These results indicated that Xi Lei San could significantly ameliorate the histological features of colitis in rats induced with DSS.

### 3.3. Xi Lei San Prevented the Activation of NLRP3 Inflammasomes and Autophagy in Rats with DSS-Induced Colitis

**Figure 3:**
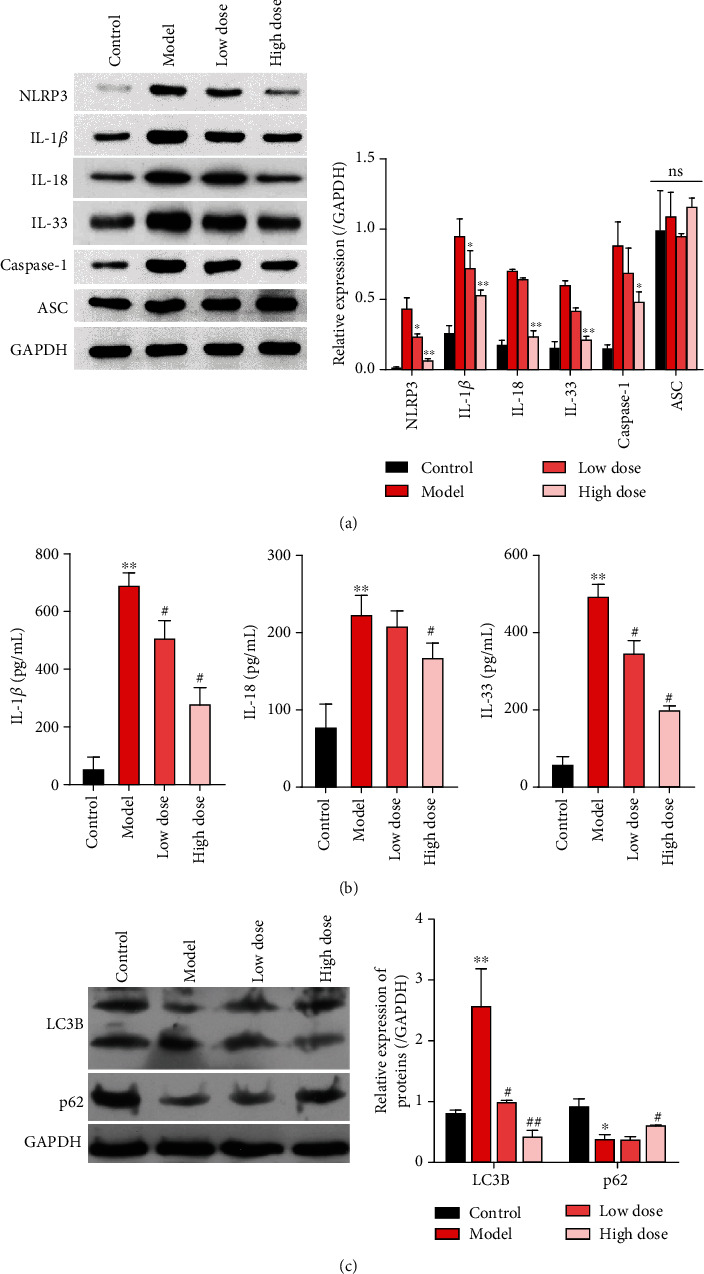
Xi Lei San prevented the activation of NLRP3 inflammasomes and autophagy in rats with DSS-induced colitis. (a) The DSS model rats with ulcerative colitis were treated with low or high doses of Xi Lei San. NLRP3, IL-1*β*, caspase-1, and ASC expression determined by western blotting. GAPDH served an internal reference protein. (b) ELISA kits were used to analyze the concentrations of IL-1*β*, IL-18, and IL-33 in each group. (c) Autophagy-related proteins, LC3B and p62, were measured by western blotting. ^∗^*P* < 0.05 and ^∗∗^*P* < 0.01 vs. the control group; ^#^*P* < 0.05 and *^##^P* < 0.01 vs. the model group.

We next sought to examine how Xi Lei San affected inflammation in rats with colitis induced by DSS. First, the expression levels of NLRP3 inflammasome-related proteins were determined by western blotting. Our results showed that the levels of NLRP3, IL-1*β*, and caspase-1 expression were significantly increased in the DSS-induced model group when compared with those in the control group, and those elevated expression levels could be significantly reduced by Xi Lei San treatment and particularly by treatment with high doses of Xi Lei San. In contrast, there was little change in the levels of ASC in each group (Figure 3(a)). ELISA data showed that the DSS model rats had significantly higher levels of IL-1*β*, IL-18, and IL-33 in their serum, and those increases could be reversed by Xi Lei San, with the high-dose Xi Lei San group showing the more significant reversal effect (*P* < 0.05 and *P* < 0.01, Figure 3(b)). As shown in Figure 3(c), activation of autophagy in DSS model rats was presented as characterized by higher value of LC3B II/I and weaker expression of p62, while the LC3B II/I ratio was reversed by Xi Lei San delivery, together with elevated p62 expression (Figure 3(c)). When taken together, these findings showed that Xi Lei San significantly inhibited the activity of NLRP3 inflammasomes and autophagy in the DSS-induced colitis model rats.

### 3.4. Xi Lei San Inhibited NLRP3 Inflammasomes and Apoptosis and Reduced ROS and Inflammatory Cytokine Production in TNF-*α*-Stimulated CACO2 Cells

**Figure 4:**
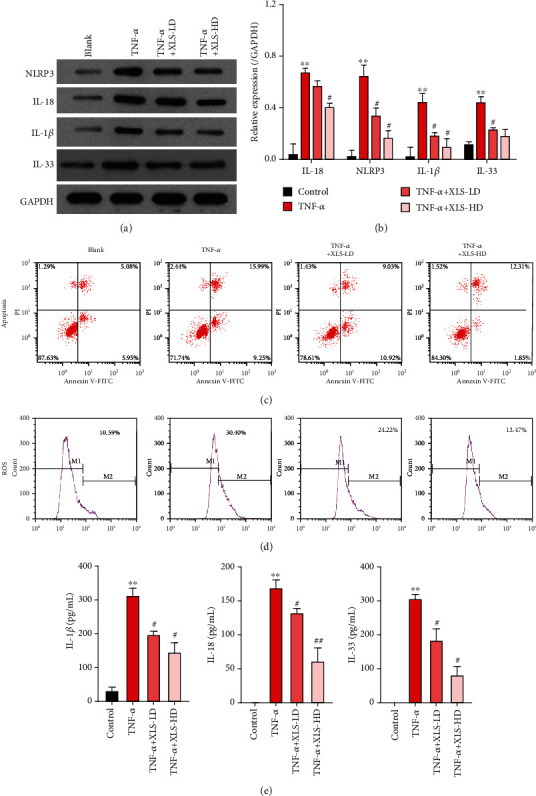
Xi Lei San inhibited NLRP3 inflammasome activity and apoptosis and reduced the levels of ROS and inflammatory cytokines in TNF-*α*-stimulated CACO2 cells. A cellular model of colitis was constructed by inducing CACO2 cells with TNF-*α*, and groups of colitis model cells were then treated with low or high doses of Xi Lei San, respectively. (a) NLRP3, IL-18, IL-33, and IL-1*β* levels in the CACO2 cells were examined by western blotting. (b) A statistical analysis of western blotting results was performed based on grayscales. (c) The effect of Xi Lei San on the apoptosis of TNF-*α*-induced CACO2 cells was examined by flow cytometry. (d) The ROS levels in each group of CACO2 cells were also examined by flow cytometry. (e) ELISA was performed to monitor IL-1*β*, IL-18, and IL-33 levels. ^∗∗^*P* < 0.01 vs. the control group; ^#^*P* < 0.05 and ^##^*P* < 0.01 vs. the TNF-*α* group.

Similarly, *in vitro* experiments further verified the effects of Xi Lei San on NLRP3 inflammasomes, apoptosis, ROS, and inflammatory factors. For conducting these studies, we established a cellular model of colitis by stimulating CACO2 cells with TNF-*α*. Our results showed that the levels of NLRP3, IL-18, IL-33, and IL-1*β* expression in the TNF-*α*-induced model cells were significantly higher than those in blank cells. Furthermore, administration of Xi Lei San could partially reverse the increases in those three proteins in TNF-*α*-stimulated CACO2 cells (*P* < 0.05 and *P* < 0.01, Figures 4(a) and 4(b)). We also found that the apoptosis rate of CACO2 cells in the TNF-*α* induction group was higher than that of cells in the blank group, and the enhancement in apoptosis could be attenuated by Xi Lei San (Figure 4(c)). Furthermore, we found that TNF-*α* could increase the levels of ROS in CACO cells, while Xi Lei San attenuated those increases (Figure 4(d)). Finally, the levels of IL-1*β*, IL-18, and IL-33 were significantly higher in the TNF-*α* model cells than those in the control cells, while those levels were significantly lower in the Xi Lei San-treated cells than in the TNF-*α*-treated cells (*P* < 0.05 and *P* < 0.01, Figure 4(e)). These data further confirmed that Xi Lei San could exert a regulatory effect in an *in vitro* CACO2 cell model of colitis.

### 3.5. Xi Lei San Suppressed Apoptosis, ROS Production, and Autophagy in TNF-*α*-Stimulated CACO2 Cells by NLRP3 Inflammasomes

**Figure 5:**
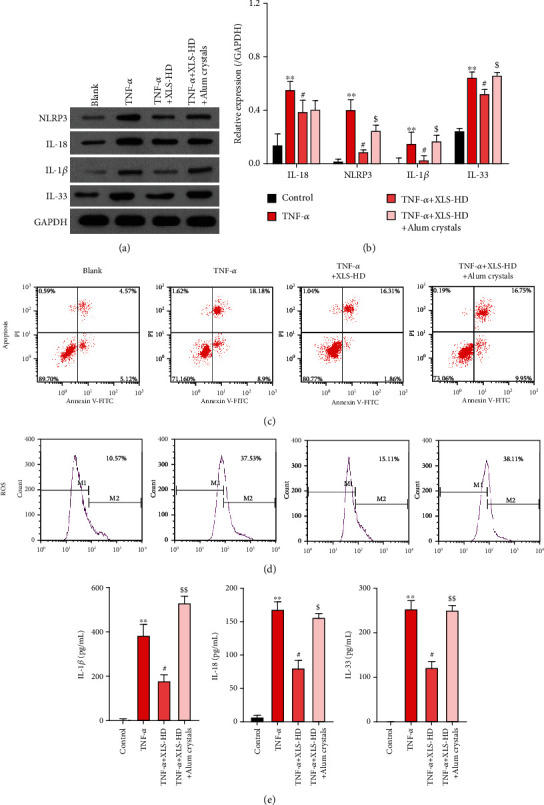
Xi Lei San suppressed apoptosis and reduced ROS levels and inflammation in TNF-*α*-stimulated CACO2 cells by inhibiting NLRP3 inflammasomes. TNF-*α*-induced CACO2 cells were treated with Xi Lei San and alum crystals. (a) A western blotting analysis was performed to examine the levels of NLRP3, IL-18, IL-33, and IL-1*β* expression in the CACO2 cells following their treatment with Xi Lei San and alum crystals. (b) A quantitative analysis of western blotting results was performed. (c) A flow cytometry analysis showed the apoptosis rate of CACO2 cells after treatment. (d) ROS production was determined by flow cytometry. (e) ELISA assay was used to detect the concentrations of IL-1*β*, IL-18, and IL-33 in the treated CACO2 cells. ^∗∗^*P* < 0.01 vs. the control group; ^#^*P* < 0.05 vs. the TNF-*α* group; ^$^*P* < 0.05 and ^$$^*P* < 0.01 vs. the TNF-*α*+Xi Lei San group.

**Figure 6:**
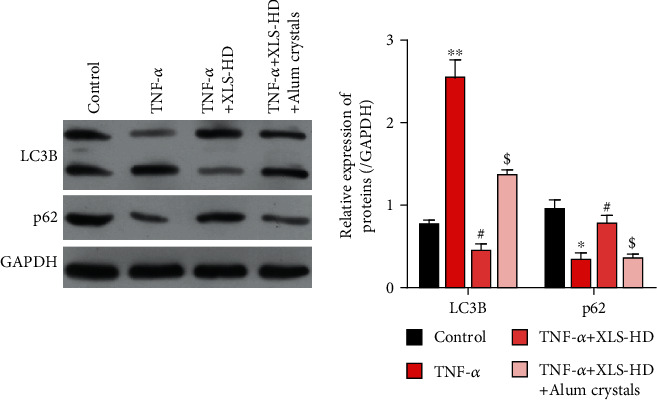
Xi Lei San reversed autophagy in rats with DSS-induced colitis. ^∗^*P* < 0.05 and ^∗∗^*P* < 0.01 vs. the control group; ^#^*P* < 0.05 vs. the TNF-*α* group; ^$^*P* < 0.05 vs. the TNF-*α*+Xi Lei San group.

After considering the relationships found among NLRP3 inflammasomes, apoptosis, ROS, and inflammation in previous studies [26, 27], we decided to further examine whether NLRP3 inflammasomes played a dominant role in the regulatory effects of Xi Lei San on apoptosis, ROS production, and inflammation in our cellular model of colitis. The colitis model cells were induced with TNF-*α* and then treated with Xi Lei San and alum crystals. As expected, the alum crystals further reversed the downregulation of NLRP3, IL-18, IL-33, and IL-1*β* expression that was mediated by Xi Lei San in TNF-*α*-stimulated CACO2 cells (*P* < 0.05 and *P* < 0.01, Figures 5(a) and 5(b)). Similarly, the inhibitory effect of Xi Lei San on apoptosis in TNF-*α*-induced CACO2 cells was noticeably reversed by the alum crystals (Figure 5(c)). We also found that alum crystals could significantly attenuate the reduction in ROS levels that was caused by Xi Lei San in TNF-*α*-stimulated CACO2 cells (Figure 5(d)). Moreover, ELISA results showed that the Xi Lei San-mediated reductions in IL-1*β*, IL-18, and IL-33 concentrations could be largely recovered by treatment with alum crystals (*P* < 0.05 and *P* < 0.01, Figure 5(e)). As shown in Figure 6, the LC3B II/I ratio was increased by TNF-*α*, along with decreased p62 expression. Xi Lei San suppressed autophagy significantly by attenuating LC3B II/I. However, LC3B II/I was reversed by alum crystal treatment, with a decreased p62 expression. When taken together, these results showed that the attenuating effects of Xi Lei San on cell apoptosis, ROS production, and inflammation could be achieved by regulating NLRP3 inflammasomes.

## 4. Discussion

The incidence of IBD has been increasing on a yearly basis, and IBD has become a universal disease of the digestive system [28]. IBD has been reported to be associated with a variety of factors, including genetic factors, the autoimmune system, gut microbes, environmental factors, and mental factors [29, 30]. Because there is no safe and effective clinical treatment for IBD, it is important to further study its pathogenesis. DSS is a water-soluble sulfated polysaccharide that has been widely used to create rat models of colitis [31]. It was found that DSS-induced colitis was particularly similar to human IBD in terms of its clinical manifestations and histopathology [32]. In our study, we used DSS to establish a rat model of colitis. We found that rats in the DSS-induced colitis group lost significantly more weight and their colon tissues showed signs of structural damage and severe inflammation when compared to colon tissues in control rats. TNF-*α*, as the initiating molecule in the cytokine network, is mainly produced by activated mononuclear macrophages [33, 34]. Research has shown that TNF-*α* can induce the production of inflammatory cytokines, amplify inflammatory chain reactions, and cause intestinal mucosal damage when participating in the inflammatory and immune responses of IBD [35, 36]. In our study, TNF-*α*-induced CACO2 cells were used as an *in vitro* model of colitis. We found that apoptosis, ROS levels, and inflammation were all significantly enhanced in the TNF-*α*-induced colitis model cells.

From the perspective of traditional medicine, IBD belongs in the category of “intestinal apnea” and “dysentery” and should be treated with drugs that clear heat, cool blood, and promote granulation [37]. Xi Lei San is composed of seven TCMs, which have the effects of detoxification, resolving stagnation, and promoting granulation [17]. Xi Lei San was originally used to treat mouth ulcers, throat erosion, and swelling [16]. The intestinal mucosa of IBD lesions show signs of ulceration, erosion, and bleeding, and the pathogenesis and manifestations of IBD lesions are similar to those of oral ulcers and laryngeal erosion. The indigo naturalis contained in Xi Lei San can cool blood detoxification, sterilize tissues, and diminish inflammation [38]. Calculus bovis factitius functions as a sedative, acesodyne, sterilizing agent, anti-inflammatory agent, and vasoconstrictor and also increases blood hemoglobin levels [39]. Borneol can relieve swelling and pain, enhance antisepsis activity, and promote tissue regeneration [40]. Pearl and ivory crumb have heat clearing and detoxification effects; they also promote granulation, the healing of mucosal ulcers, and the removal of toxins [41]. Studies have shown that Xi Lei San can relieve symptoms such as diarrhea, pus and blood in stools, and bellyache as well as promote the healing of ulcers, improve the clinical symptoms of patients, and reduce the recurrence rate of IBD [16]. In our study, we further verified that Xi Lei San could dramatically increase the body weight of rats and improve the structure and inflammation of colonic tissues in DSS-induced colitis model rats. Inflammasomes comprise a class of protein complexes that can recognize different damage signals [42]. The NLRP3 inflammasome is the main type of NLRP inflammasome and consists of NLRP3, ASC, and caspase-1 [20]. The NLRP inflammasome is an intracellular receptor that can regulate an inflammatory response [43]. NLRP3 inflammasomes, as complexes of multiple proteins, mainly regulate caspase-1 activity to promote the maturation and secretion of IL-1*β* and IL-18 and thus help to regulate the innate immune response. NLRP3 inflammasomes are also known to be associated with IBD pathogenesis [22, 44]. In our study, we found that Xi Lei San could significantly inhibit NLRP3 inflammasome activity in rats with DSS-induced colitis and also in TNF-*α*-induced CACO2 cells. We also showed that Xi Lei San could reduce apoptosis, ROS production, and inflammatory cytokine production in TNF-*α*-induced model cells by regulating NLRP3 inflammasomes. However, ASC expression was not altered by Xi Lei San administration. Apoptosis-related speck protein (ASC) is an adaptor protein that connects intracytoplasmic receptors and caspase-1 in inflammasomes. The dimer formed by ASC aggregation during inflammasome activation is referred to as ASC speck and usually exists in a soluble form. ASC is located in the cytoplasm and nucleus of cells undergoing a stress response. ASC was gathered forming dimer, as a caspase-1 activation platform [45]. The previous results reported by Samir et al. [46] are similar to ours [46]. Besides, we know that an interaction between NLRP3 inflammasome and autophagy has been widely studied. Autophagy inhibits NLRP3 inflammasome by cleaning infection pathogenic microorganism (Shao, 2019 #44) and thus enhances the innate immunity response (Jin, 2020 #50). However, evidence indicates that autophagy is positively regulated by NLRP3 inflammasomes (Dupont, 2011 #51). In this study, we found that NLRP3 inflammasomes were activated in DSS-induced rat and TNF-*α*-treated cells in vitro, along with activated autophagy. Results of this in vitro experiment hinted that NLRP3 activation by alum crystals also enhanced autophagy. This might be due to the fact that autophagy was also alum crystal treatment in vitro and this should be further focused on.

In summary, our *in vivo* and *in vitro* studies showed that Xi Lei San could significantly relieve the symptoms of colitis in DSS-induced colitis model rats by suppressing the activity of NLRP3 inflammasomes and reducing autophagy. These findings suggest Xi Lei San as a Chinese medicine that is capable of attenuating inflammation in IBD via a mechanism that involves NLRP3 inflammasomes. However, the interaction between autophagy and NLRP3 inflammasomes should be further investigated in the future.

## Data Availability

The original contributions presented in the study are included in the article; further inquiries can be directed to the corresponding author.
